# A novel strategy to map a locus associated with flowering time in canola (*Brassica napus* L.)

**DOI:** 10.1007/s00438-024-02191-w

**Published:** 2024-10-08

**Authors:** Yunming Long, Puying Zheng, James V. Anderson, David P. Horvath, Jinita Sthapit, Xuehui Li, Mukhlesur Rahman, Wun S. Chao

**Affiliations:** 1 Department of Plant Sciences, North Dakota State University, Dept. 7670, PO Box 6050, Fargo, ND 58108-6050 USA; 2 USDA/ARS, Weed and Insect Biology Research Unit, Edward T. Schafer Agricultural Research Center, 1616 Albrecht Blvd. N., Fargo, ND 58102-2765 USA

**Keywords:** Canola, Flowering time, Genome mapping, Introgression line

## Abstract

Flowering time is an important agronomic trait for canola breeders, as it provides growers with options for minimizing exposure to heat stress during flowering and to more effectively utilize soil moisture. Plants have evolved various systems to control seasonal rhythms in reproductive phenology including an internal circadian clock that responds to environmental signals. In this study, we used canola cultivar ‘Westar’ as a recurrent parent and canola cultivar ‘Surpass 400’ as the donor parent to generate a chromosome segment substitution line (CSSL) and to map a flowering time locus on chromosome A10 using molecular marker-assisted selection. This CSSL contains an introgressed 4.6 mega-bases (Mb) segment (between 13 and 17.6 Mb) of Surpass 400, which substantially delayed flowering compared with Westar. To map flowering time gene(s) within this locus, eight introgression lines (ILs) were developed carrying a series of different lengths of introgressed chromosome A10 segments using five co-dominant polymorphic markers located at 13.5, 14.0, 14.5, 15.0, 15.5, and 16.0 Mb. Eight ILs were crossed with Westar reciprocally and flowering time of resultant 16 F_1_ hybrids and parents were evaluated in a greenhouse (2021 and 2022). Four ILs (IL005, IL017, IL035, and IL013) showed delayed flowering compared to Westar (*P* < 0.0001), and their reciprocal crosses displayed a phenotype intermediate in flowering time of both homozygote parents. These results indicated that flowering time is partial or incomplete dominance, and the flowering time locus mapped within a 1 Mb region between two co-dominant polymorphic markers at 14.5–15.5 Mb on chromosome A10. The flowering time locus was delineated to be between 14.60 and 15.5 Mb based on genotypic data at the crossover site, and candidate genes within this region are associated with flowering time in canola and/or Arabidopsis. The co-dominant markers identified on chromosome A10 should be useful for marker assisted selection in breeding programs but will need to be validated to other breeding populations or germplasm accessions of canola.

**Supplementary Information:**

The online version contains supplementary material available at 10.1007/s00438-024-02191-w.

## Introduction

Canola-rapeseed (*Brassica napus* L.) is an important oilseed crop, and global demand for canola oil production is increasing steadily. Flowering time in rapeseed is known to play a vital role in seed yield and environmental adaptation (Wang et al. 2011). However, the environmental impacts of climate change, such as increased episodes of heat and drought stress, on canola oilseed yields appear to be greatest during the flowering period (Secchi et al. 2023) and, thus, flowering time is a major target for canola-rapeseed breeders. Gaining additional knowledge on genetic factors associated with flowering time could provide new approaches for breeders to improve canola-rapeseed germplasm that allow growers to minimize the environmental impacts of climate change on oilseed production and oil quality.

Flowering time is a trait known to impact plant fitness and crop yield, and plants have evolved systems to control their seasonality in reproductive phenology via internal timekeepers (or circadian clock) and responses to environmental signals (Gaudinier and Blackman 2020). Flowering is induced by two environmental stimuli, photoperiod (daylight) and temperature (vernalization) in temperate climates (Andrés and Coupland 2012). Plants can be classified into three photoperiodic groups: short-photoperiod, long-photoperiod, and photoperiod-neutral plants (Garner and Allard 1920). Short-photoperiod plants bloom when the daylight hours are short, which usually occurs in the early spring or early fall. Long-photoperiod plants bloom when the daylight hours are long, which usually occurs in late spring and summer. Day-neutral plants flower irrespective of the photoperiodic conditions. A mechanism for photoperiodic time measurement is present in leaves. For example, Arabidopsis (*Arabidopsis thaliana*) is a long-photoperiod plant that perceives increased photoperiod through a mechanism that transcriptionally and post-transcriptionally upregulates the transcription factor CONSTANS (CO). When CO levels reach a threshold, it activates the expression of *FLOWERING LOCUS T* (*FT*), which encodes a mobile hormonal signal known as florigen that is transported from the leaf to the shoot apex to induce flowering (Andrés and Coupland 2012; Johansson and Staiger 2015). In contrast, short-photoperiod conditions lead to low levels of *FT* expression and delays in flowering (Kobayashi et al. 1999).

Winter annuals and biennials require extended exposure to cold temperatures or vernalization to induce flowering in the spring; this is a mechanism that plants have evolved to prevent precocious flowering in the fall (Bouché et al. 2017). These plants have developed repression mechanisms that block the transition from vegetative to reproductive growth in the fall; however, vernalization can overcome the repressive processes. In Arabidopsis, most of the natural diversity of the vernalization requirement can be attributed to allelic variation at *FRIGIDA* (*FRI*) and its regulation of the MADS-box gene *FLOWERING LOCUS C* (*FLC*), which is expressed before exposure to cold but is repressed stably upon cold exposure. FLOWERING LOCUS C represses flowering by inhibiting the transcription of *FT* in leaves and *SUPPRESSOR OF OVEREXPRESSION OF CO1* (*SOC1*) in the shoot apical meristem (Michaels and Amasino 1999; Hepworth et al. 2002; Helliwell et al. 2006). Other vernalization-related, cold-induced key alterations include the expression of antisense *FLC* transcripts, collectively named COOLAIR (Swiezewski et al. 2009; Castaings et al. 2014; Marquardt et al. 2014) and the expression of a gene encoding VERNALIZATION INSENSITIVE 3, which plays a role in the stable repression of *FLC* (Sung and Amasino 2004) in Arabidopsis.

Components important for crop yield include the number of pods/plant, the number of seeds/pod and the mean individual seed weight, in which pod number is the most important factor as it is affected largely by environmental constraints (Diepenbrock 2000; Faraji 2012; Kirkegaard et al. 2018). Because the flowering period in rapeseed can take 2–6 weeks (Gan et al. 2016; Kirkegaard et al. 2018) and the flowers produced in the first 2–3 weeks from anthesis contribute to 75% of the pods at maturation (Tayo and Morgan 1975), optimal flowering time is essential in determining final seed yield (Tayo and Morgan 1975; Diepenbrock 2000; Faraji 2012; Kirkegaard et al. 2018; Zhang et al. 2021). Genomics studies have identified many candidate genes involved in the flowering network, vernalization process, and photoperiod pathway (Raman et al. 2016; Xu et al. 2016; Schiessl et al. 2015; Vollrath et al. 2021). These findings imply that the regulation of flowering time is a complex process in *B. napus*, as in the well-studied model plant *A. thaliana* (Fornara et al. 2010; Bouché et al. 2016), and controlled by many loci on different chromosomes. However, the genetic mechanism of flowering time in *B. napus* remains to be elucidated.

Mapping the genetic basis of agronomic traits is critical to revealing the biological mechanisms that underlie plant phenotypes, and genetic mapping and physical mapping are two general methods of genome mapping. The effects of flowering time and heterosis were observed in a canola hybrid between Westar and Surpass 400 during a genomic mapping study on blackleg resistance in canola (Long et al. 2011), and the loci responsible for these two traits are located around 14.5 Mb of chromosome A10 of Surpass 400. Two known polymorphic markers, M779 and M781, were used to select the target DNA segment from Surpass 400, and a chromosome segment substitution line (CSSL) was developed; this CSSL contains a 4.6 Mb introgressed DNA segment from chromosome A10 of Surpass 400 in the genetic background of Westar genotype. In addition, eight introgression lines (ILs) were developed carrying a series of different lengths of the 4.6 Mb introgressed chromosome segment for mapping of the flowering time locus. A locus which explained the phenotypic variation in flowering time was mapped to the 1 Mb genomic DNA region in chromosome A10 of Surpass 400. The focus of this report is to introduce this novel mapping strategy and to describe those causative candidate genes regulating flowering time which may help the breeding of flowering time traits in rapeseed.

## Materials and methods

### Plant materials

Two canola populations were used in this study; one was a chromosome segment substitution line (CSSL) population, and the other was an introgression line (IL) population. The CSSL population was derived from the cross between the canola cultivars Westar and Surpass 400, and the IL population was derived from the cross between a Westar and a CSSL. The details for developing these two populations are described below. Westar also named Westar summer rape (*Brassica napus* L.) was developed at the Agriculture Canada Research Station, Saskatoon, Saskatchewan and licensed in 1982; it has low erucic acid (0.2%) and glucosinolate (15 pmol g meal^−l^) content in the seed. Westar seeds were obtained from North Dakota State University. Surpass 400 (*Brassica napus* L.) is a blackleg resistant spring-type cultivar, and the seeds were obtained from the Pacific Seeds (Toowoomba, Australia).

### DNA extraction

Genomic DNAs of all lines including a Westar, a Surpass 400, a chromosome segment substitution line (CSSL), hybrids (Westar♀ × Surpass 400♂ and Westar♀ × CSSL♂), backcrossed offsprings, and introgression lines (ILs) were extracted from young leaves using CTAB method (Doyle and Doyle 1990). Ground leaves were mixed with extraction buffer containing 1.4 M NaCl, 100 mM Tris HCl pH 8.0, 2% polyvinylpyrrolidone (PVP), 2% (w/v) CTAB, 20 mM EDTA pH 8.0, and 0.1 M β-mercaptoethanol and then incubated at 65 °C for 30 min. DNA was precipitated in 35% isopropanol and 0.5 M salt, and the resulting pellet was washed twice in 75% ethanol and dried in a Savant SpeedVac® Concentrator. DNA samples were dissolved in 200 μl distilled deionized water, and concentrations were measured using a NanoDrop™ spectrophotometer. DNA samples were diluted to 30–50 ng/μl before use.

### PCR reaction and amplicon detection

Approximately 30–50 ng/μl of genomic DNA was used for DNA amplification. PCR reaction was performed on the GeneAmp PCR System 9700 (Applied Biosystems, Foster City, CA) and BIO-RAD C1000TM Thermal Cycler 9600. For SSR and Indel markers, DNA amplification was carried out in a final 10-μl volume of reaction containing 1× PCR buffer, 100 μM dNTP, 0.1–0.2 μM primers, 2 μl genomic DNA, and 1 unit of Taq DNA polymerase. PCR program was initially incubated at 94 °C for 3 min, followed by 33 cycles at 94 °C for 40 s, 55 °C for 60 s, and 72 °C for 60 s before ending with a final extension at 72 °C for 5 min and cool down at 10 °C. For semi-thermal asymmetric reverse PCR (STARP) markers, DNA amplification was carried out in a total volume of 10 μl mixture using a touchdown program according to Long et al. (2016). The 10 μl reaction mixture contains 1× PCR buffer, 50 μM of each dNTP, 200 nM common reverse primer, 200 nM of each of priming element-adjustable primers (PEA-primer 1 and PEA-primer 2), 1 U of Taq DNA polymerase, and 2 μl genomic DNA. PCR was performed with initial denaturation at 94 °C for 3 min, followed by 4 cycles touchdown PCR program at 54 °C annealing temperature with a decrease of 1 °C per cycle and 32–36 cycles of normal PCR ending with an extension step at 62 °C for 2 min. The PCR products were visualized on 6.5% polyacrylamide using a Licor 4200 automated DNA sequencer (Manufacturer: LI-COR).

### Development of a chromosome segment substitution line (CSSL)

A CSSL was developed based on marker assisted selection to introgress a 4.6 Mb DNA segment from chromosome A10 of Surpass 400 to Westar. In this work, SSR markers were used for target region selection to select lines containing 13–17.6 Mb DNA segment from chromosome A10 of Surpass 400. SSR markers were also used for background selection to expedite in recovery of recurrent parent, Westar. The procedure is described as follows. Initially, a total of 40 SSR markers were tested, and 18 out of 40 markers showed polymorphism between Westar and Surpass 400 (Supplemental Table S1, Spreadsheet ‘40 SSR markers’; polymorphic markers are highlighted in yellow). Two of these polymorphic markers, M779 and M781, that are localized around the target locus at 14.5 Mb of chromosome A10 were used to select target DNA segment from chromosome A10 of Surpass 400 (target region selection). To obtain near isogenic lines, we first used 4 markers (M786, M789, M800, and M817) to genotype 8 × 96 BC1F1 plants and obtained 33 plants showing homozygous for Westar alleles (Supplemental Table 1, Spreadsheet ‘Screening BC1 population’). Four more markers (M780, M804, M812, and M819) were used to genotype these 33 plants, and two plants (#441 and #601) were selected that showed homozygous for Westar alleles (M804 and M812) and heterozygous for M780 (Supplemental Table 1, Spreadsheet ‘Genotyping 33 BC1 plants’). These two plants were then genotyped using all 18 polymorphic markers, and the one (#601) that showed minimum Surpass 400 background was selected and used to backcross to Westar for the generation of BC2F1 population (Supplemental Table 1, Spreadsheet ‘Genotyping 33 BC1 plants’).

To screen BC2 population, we first tested 236 new SSR markers for polymorphism among Westar, #601, and Surpass 400, and 19 polymorphic markers were obtained (Supplemental Table 1, Spreadsheet ‘236 SSR markers’; polymorphic markers were highlighted in yellow). Two of these polymorphic markers (M7 and M95) were used to genotype 2 × 96 BC2F1 plants, and 40 plants were obtained that showed homozygous for Westar alleles. We then genotyped these 40 plants using 2 other markers (M779 and M105), and 11 plants that showed homozygous for Westar alleles and heterozygous for M779 were selected. These 11 plants were genotyped using 8 markers (M26, M62, M126, M151, M152, M218, M226, and M781), and 4 plants (601-26, 601-50, 601-62, and 601-114) were selected (Supplemental Table 1, Spreadsheet ‘Screening BC2 population’) and further genotyped with 41 markers. One plant (601-26) that showed minimum Surpass 400 background was selected and used to backcross with Westar for the generation of BC3F1 population.

To screen BC3F1 population, we first used 3 markers (M62, M780, and M781; M62 marker locus is at the linkage group C4, and M780 and M781 are localized around target heterotic locus at 13.0 and 14.5 Mb of chromosome A10, respectively) to genotype 2 × 96 BC3F1 plants and obtained 8 plants that were homozygous for Westar alleles (M62 and M781) and heterozygous for M780 alleles. We then genotyped these 8 plants using 12 other markers (Supplemental Table 1, Spreadsheet ‘Screening BC3 population’), and one plant (14-04-32) that showed minimum Surpass 400 background was selected. BC3F2 seeds were generated by self-pollination of 14-04-32 ⊗ . Forty-eight individual BC3F2 seedlings were genotyped with 2 markers (M779 and M780) and one homozygous plant (14-04-32-26) was selected. This homozygous near isogenic line was designated as Chromosome Segment Substitution Line (CSSL) which contains A10 segment from Surpass 400 with the genetic background of Westar and was used to develop introgression lines (ILs).

### Development of introgression lines (ILs)

Marker-assisted strategy was used to develop ILs carrying a series of the introgressed DNA in chromosome A10 of Surpass 400 for mapping various traits. To identify as many crossover sites between 13.0 and 17.6 Mb of chromosome A10, 21 SSR and 6 SNP markers were developed that across every 500 Kb chromosome region (Supplemental Table 1, Spreadsheet ‘New SSR and STARP markers’). Ten polymorphic co-dominant markers were obtained. These co-dominant markers were used to develop 8 ILs. F2 population was generated by selfing a F1 hybrid (Westar♀ × CSSL♂). Seedlings (4 × 96 F2 individuals) were first genotyped to determine recombinants using SSR markers M780, M862, and M868, and 96 plants were identified to show crossover events (Supplemental Table 1, Spreadsheet’96 recombinants’). These 96 recombinants were further genotyped using markers M876 (STARP), M862 (SSR), M780 (SSR), M878B (STARP), M879RB (SSR), and M868 (SSR) at 13.5, 14.0, 14.5, 15.0, 15.5, and 16.0 Mb position on chromosome A10, respectively, and they were organized into 8 groups (Supplemental Table 1, Spreadsheet ‘8 groups of recombinants’). The recombinants in each group carried different lengths of introgressed chromosome segments in A10 of Surpass 400 with the genetic background of Westar genotype. One recombinant was randomly selected from each group and self-pollinated to generate F3 families, and eight homozygous recombinant ILs were selected using markers M876 (STARP), M877 (STARP), M862 (SSR), M780 (SSR), M892 (SSR), and M868 (SSR) (Supplemental Table S1, Spreadsheet ‘8 ILs’).

### Flowering time studies

Three-gallon pots were prepared as follows: One part of sandy loam (S&S Landscaping) was mixed with 2 parts of ProMix (Premier Horticulture, Inc., Quakertown, PA [Tessman’s]) in a 3 Cubic-Feet Electric Cement Mixer (Fargo Rentall). The pots were placed with paper towel at the bottom, filled with soil mix, tamped lightly, and loaded on greenhouse tables. Each 3-gallon pot was top-dressed with slow-release fertilizer (Multicote) at 3 tablespoons per pot, planted 3–5 seeds per pot at ~0.5 inch below the surface, and watered gently and thoroughly. At 1 week, seedlings were thinned to 1 plant per pot. Flowering time (initiation and termination of flowering) was recorded during plant development (Supplemental Table S2). There was a total of 28 genotypes (16 IL × Westar reciprocal hybrids, 8 ILs, 2 CSSL × Westar reciprocal hybrids, 1 CSSL, 1 Westar). The experiments were performed using randomized complete block design and were repeated 4 times (3 replicates per experiment) in 2 years.

### Genotyping

Westar, CSSL, and IL013 were genotyped using genotyping-by-sequencing (GBS) according to the procedure described in Chao et al. (2023). Briefly, the GBS library was constructed using the same protocol described in Horvath et al. (2020). The GBS library was sequenced on an Illumina (San Diego, CA) HiSeq 4000 to generate single-end, 100-bp reads at the Genomic Sequencing and Analysis Facility, University of Texas Southwestern Medical Center, Dallas, TX. The SNP discovery and genotype calling was performed with the TASSEL-GBS pipeline (Glaubitz et al. 2014) using the Brassica napus v4.1 (Chalhoub et al. 2014) as reference genome.

### Selection of candidate flowering genes

The regions delimiting the introgressed region was pulled from the brassica napus v5 gff3 file (downloaded from https://www.genoscope.cns.fr/brassicanapus/data/Brassica_napus.annotation_v5.gff3.gz that was further annotated by mapping the gene functions to the gff file). The resulting region was scanned for genes associated with flowering time.

### Statistical data analysis

Statistical analysis was performed in JMP Pro v. 17.0.0 (SAS Institute, Cary, North Carolina, USA). One-way analysis of variance (ANOVA) of flowering time by canola lines and crosses were performed followed by Tukey–Kramer HSD to test significant differences between the means (Supplemental Table S3).

## Results and discussion

### Mapping of flowering time locus by Marker-Assisted Lines Development with Series of Introgressed Chromosome Segments (MALDSICS)

**Fig. 1:**
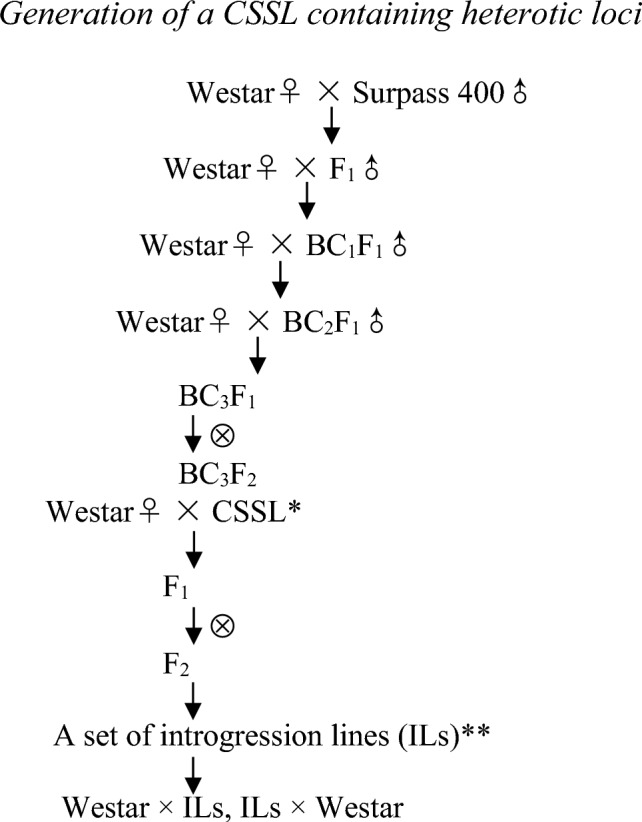
Development of a CSSL and 8 ILs. The procedure of developing a CSSL is briefly described as follows. First, F1 seeds were produced from a cross between Westar♀ and Surpass 400♂. BC1 F1 seeds were produced from a cross between Westar♀ and F1♂. BC1F1 seedlings were genotyped using SSR markers, and one desired BC1F1 genotype (601) was obtained. BC2F1 seeds were produced from a cross between Westar♀ and the selected BC1F1 genotype (601♂). BC2F1 seedlings were genotyped and one desired BC2F1 genotype (601-26) was obtained. BC3F1 seeds were produced from a cross between Westar♀ and the selected BC2F1 genotype (601-26♂). BC3F1 seedlings were genotyped and one desired BC3F1 genotype (14-04-32) was obtained. BC3F2 seeds were generated by self-pollination of this genotype (14-04-32 ⊗). A homozygous plant (14-04-32-26) was obtained after genotyping BC3F2 seedlings. To develop 8 ILs, F2 population was generated by selfing a F1 hybrid (Westar♀ × CSSL♂). Seedlings were genotyped to determine recombinants, and 96 plants were identified to show crossover events. These 96 recombinants were self-pollinated to generate F3 families, and homozygous recombinant ILs were determined by marker assisted selection from some F3 families. Eight ILs were developed carrying a series of introgressed chromosome segments in A10 of Surpass 400 using five co-dominant polymorphic markers at 13.5 (M877), 14.0 (M862/M14-2), 14.5 (M780), 15.0 (M892), and 16.0 (M868/M16-2) Mb chromosome position. * The chromosome segment substitution lines (CSSLs) were homozygous lines selected from BC3F2 population. These lines contain a chromosome A10 single-segment of Surpass 400 with the genetic background of Westar. ** Introgression lines (ILs) were homozygous lines carrying a series of introgressed chromosome single-segments from the CSSL

**Fig. 2:**
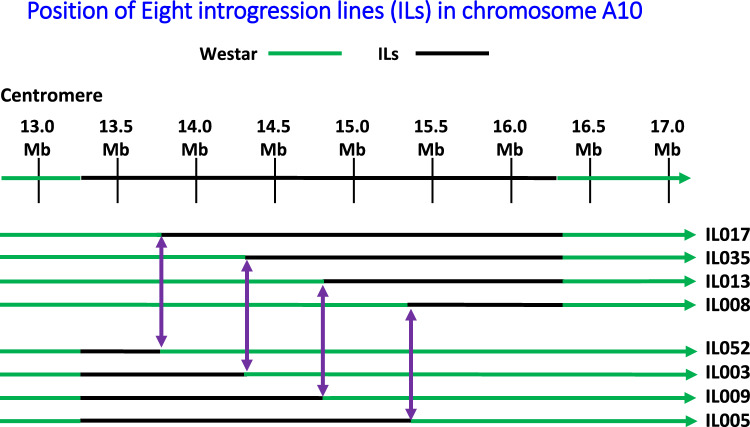
Molecular mapping of the flowering gene by generating ILs using co-dominant polymorphic markers. This figure shows chromosome arrangements of eight ILs carrying different lengths of introgressed chromosome segments from the CSSL. Six co-dominant polymorphic markers at 13.5, 14.0, 14.5, 15.0, and 16.0 Mb position were developed on chromosome A10 and were used to determine introgressed chromosome arrangements. Purple arrows indicate markers used to determine recombinants. Green lines represent Westar genome, and black lines represent introgressed segment. In every two adjacent markers, two recombinants were randomly selected in which one carries the exotic chromosome segment at the upstream of crossover site, and the other carries the exotic chromosome segment at the downstream of crossover site

We developed a canola CSSL with the genetic background of Westar based on traditional backcross and molecular marker-assisted selection. This CSSL carries a 4.6 Mb introgressed chromosome A10 segment of Surpass 400 and has delayed flowering time trait comparing to Westar. We then adopted a MALDSICS strategy to generate eight introgression lines (ILs) carrying a series of introgressed chromosome A10 segments of Surpass 400 with different lengths for mapping of the flowering time locus. The MALDSICS is a novel mapping method used for the first time to identify flowering time loci in canola. The scheme for development of 8 ILs from a CSSL are shown in Fig. 1. The 8 homozygous recombinant ILs were selected using six different codominant markers at 13.5, 14.0, 14.5, 15.0, 15.5, and 16.0 Mb position on chromosome A10 (Supplemental Table S1, Spreadsheet ‘8 ILs’). The arrangement of introgressed chromosome segments for these 8 ILs are shown in Fig. 2. As illustrated in this figure, in every two adjacent markers, two recombinants were randomly selected in which one carries the exotic chromosome segment at the upstream of crossover site, and the other carries the exotic chromosome segment at the downstream of crossover site. This design makes mapping of target trait efficient because the flowering time locus can be quickly mapped between two adjacent markers by associating the phenotypic data with the introgressed chromosome segments.

**Fig. 3:**
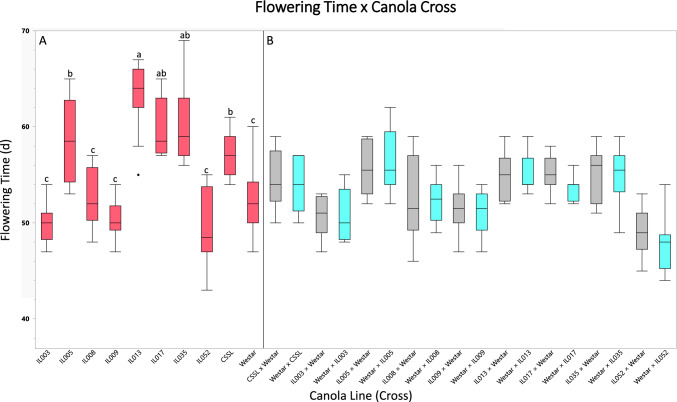
Flower times for 28 canola genotypes. The flowering times for 28 genotypes including 10 parental lines (**A**) and 18 reciprocal crosses (**B**) were determined based on their days to first open flower. The experiments were repeated 4 times in 2 years, and there were 3 replicates per genotype per experiment. Box plots present the interquartile range with the line as the median and whiskers as the minimum and maximum values. Differences in flowering time means among the parental lines were evaluated using Tukey–Kramer HSD (**A**), and the values with different letters in a column are significantly different. Differences in flowering time means among the parental lines and their reciprocal crosses (**B**) were also evaluated and presented in Supplemental Table S3 (spreadsheet ‘All genotypes’)

The hybrids along with their parents were grown in the greenhouse, and the time required to initiate first flower from the planting date was used to map the flowering time locus. A total of 28 genotypes (16 IL × Westar reciprocal hybrids, 8 ILs, 2 CSSL × Westar reciprocal hybrids, 1 CSSL, 1 Westar) were tested. The results are shown in Fig. 3 (see Supplemental Table S2 for raw data). Among 9 parental genotypes, 4 genotypes (IL003, IL008, IL009, and IL052) showed similar flowering time as the other parental line, Westar, and their first open flower was observed around 52 days after planting of seed. The reciprocal crosses of these 4 genotypes also took similar (or less) days to produce their first open flower. In contrast, IL005, IL013, IL017, IL035, and CSSL delayed flowering substantially; their first open flower was observed at 57 days (CSSL) after planting of seed to 63 days (IL13) after planting. One-way analysis of variance (ANOVA) of flowering time by canola parental lines (Fig. 3A; Supplemental Table S3) revealed significant differences in flowering time among the lines (*P* < 0.0001; Supplemental Table S3). The results indicated that these five genotypes possess the flowering locus responsible for delaying flowering time; in addition, flowering time appears to involve partial or incomplete dominance, since the reciprocal crosses of these five genotypes displayed a phenotype that is an intermediate in flowering time compared to their homozygote parents. Our results agree with the findings for garden pea (*Pisum sativum* L.) that days to flowering showed partial dominance (Abbas 2012). There were some differences in flowering duration as well, but they did not correlate with the days to flowering in this study (Coefficient of correlation = 0.21). Thus, the duration of flowering trait did not map to the introgressed region of interest. This result was somewhat surprising since a previous study indicated a correlation of 0.81 between flowering time and flowering duration (Khayat et al. 2012). This suggests that the genes impacting days to flowering in these introgression lines were not ones that also impact the duration of flowering, and that these two traits may not be completely linked.

**Fig. 4:**
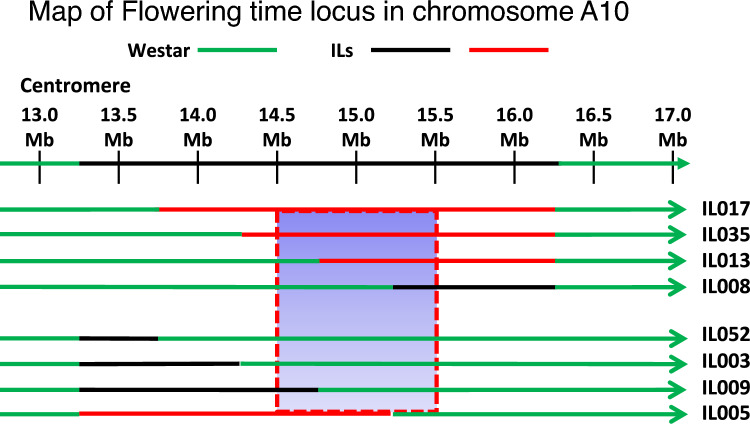
Mapping of flowering time locus. In this illustration, both red and black lines represent introgressed Surpass 400 chromosome segment, in which red color indicates that these ILs (IL005, IL013, IL017, and IL035) showed delayed flowering time compared to Westar, whereas black color indicates that these ILs (IL003, IL008, IL009, and IL052) showed similar flowering time as Westar. Based on these results, the region containing flowering locus is between 14.5 and 15.5 Mb

**Fig. 5:**
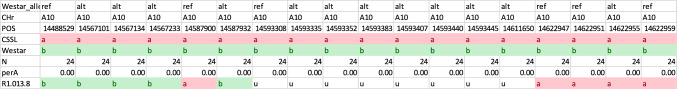
Fine mapping of flowering time locus. Genotyping data indicate that the crossover site between Waster and CSSL for IL013 was around 14.60 Mb (14,593,308–14,611,650 base pair) in chromosome A10 of Surpass 400. a and b: markers represented CSSL and Westar lines, respectively; u: markers were ambiguous in this region

Phenotypic data mentioned above was used to map the flowering time locus based on MALDSICS. As shown in Fig. 4, the locus responsible for delayed flowering is located at the genome of IL005, IL013, IL017, and IL035, but not at IL003, IL008, IL009, and IL052. The results allowed us to map the flowering time locus to be between 14.5 and 15.5 Mb (red lines within the red-dotted rectangle) in chromosome A10 of Surpass 400. In addition, IL013 has a crossover site (between Waster and CSSL) at 14.5–15.0 Mb region, and genotyping results indicated that this crossover site was at 14.60 Mb in chromosome A10 (Fig. 5; Supplemental Table S4), which further delineates the flowering time locus to be between 14.60 and 15.5 Mb.

### Underlying candidate genes associated with flowering time

This 1-Mb mapped region was found to contain a total of 260 canola gene models (Supplemental Table S5, spreadsheet ‘Gene models’), and 5 gene models were associated with flowering time (Table 1; Supplemental Table S5, spreadsheet ‘Flowering associated gene models’). Among these five gene models, a MADS-box *FLOWERING LOCUS C* (*FLC*) transcription factor was identified at 14,998,617–15,003,197 bp region on chromosome A10 of Surpass 400. MADS-box FLC plays an essential role in the regulatory network that controls flowering and functions as a major repressor of floral transition (Michaels and Amasino 1999; Sheldon et al. 1999). The expression of *FLC* is downregulated during vernalization and cold treatment (Boss et al. 2004; Taylor et al. 2017), and histone deacetylation and subsequent trimethylation at lysine 27 (H3K27) are involved in *FLC* repression (Deng et al. 2007; Zeng et al. 2020). A nearby *HISTONE ACETYLTRANSFERASE* (*HAC*) at 15,120,625–15,123,078 bp region may be involved in the regulation of flowering time via alteration of *FLC* expression. In Arabidopsis, lesions in *AtHAC1* triggered a late-flowering phenotype (Deng et al. 2007). In contrast, histone H3 trimethylation at lysine 4 (H3K4) and histone acetylation are associated with *FLC* expression (Deng et al. 2007; Jiang et al. 2011). Another nearby gene model orthologous to Arabidopsis *ARABIDOPSIS TRITHORAX-RELATED PROTEIN 5* (*ATXR5*) at 15,105,735–15,107,523 bp region may be involved in the regulation of flowering time via activation of *FLC*. Trithorax-group proteins is known to play essential regulatory roles in chromatin modification to activate transcription (Schuettengruber et al. 2007; Howe et al. 2017). *ATXR5* encodes histone methyltransferase that deposits histone H3 lysine 27 monomethylation (H3K27me1) in *Arabidopsis* chromatin (Jacob et al. 2009; Bergamin et al. 2017), and ATXR7 plays a role in histone H3K4 methylation and is required for transcriptional activation of *FLC* in *Arabidopsis* (Tamada et al. 2009). Moreover, a *NAC DOMAIN CONTAINING PROTEIN 50* (*NAC052*) and a *FLOWERING PROMOTING FACTOR 1* (*FPF1*) were identified at 14,661,715–14,662,927 and 14,817,233–14,817,562 bp, respectively, in chromosome A10. NAC052 regulates gene expression and flowering time by associating with the histone demethylase *JUMONJI14* (*JMJ14*) (Ning et al. 2015), and *FPF1* is involved in the promotion of flowering (Kania et al. 1997; Melzer et al. 1999; Wang et al. 2014). Giving the significant link between the mapped gene models and flowering time phenotype, the alteration of DNA sequence from any of above five gene models would cause a late-flowering phenotype.

**Table 1:** Flowering time associated gene models located between 14.60 and 15.5 Mb in chromosome A10 of Surpass 400

Chrm	Locus (canola)		Orientation	Gene_ID (canola)	Gene model ID	Locus (Arab)	Name
chrA10	14,661,715	14,662,927	+	BnaA10g21230D	GSBRNA2T00135820001	AT3G10480	NAC DOMAIN CONTAINING PROTEIN 50 (NAC052)
chrA10	14,817,233	14,817,562	−	BnaA10g21640D	GSBRNA2T00135871001	AT5G10625	FLOWERING PROMOTING FACTOR 1 (FPF1)
chrA10	14,998,617	15,003,197	+	BnaA10g22080D	GSBRNA2T00135921001	AT5G10140	FLC MADS-box transcription factor family protein (FLC)
chrA10	15,105,735	15,107,523	+	BnaA10g22360D	GSBRNA2T00135954001	AT5G09790	ARABIDOPSIS TRITHORAX-RELATED PROTEIN 5 (ATXR5)
chrA10	15,120,625	15,123,078	+	BnaA10g22400D	GSBRNA2T00135959001	AT5G09740	HISTONE ACETYLTRANSFERASE of the MYST family 2 (HAC)

In addition, the 96 recombinants in 8 IL groups (Supplemental Table S1, Spreadsheet ‘8 groups of recombinants’) can be used to fine map the flowering time locus and to further delineate the causative gene models associated with delayed flowering since the recombinants in each IL group carried different lengths of introgressed chromosome segments. Thus, the identification of the lengths of introgressed chromosome segments from ILs that had the crossover site between 14.5 and 15.5 Mb with delayed flowering phenotype may delineate the flowering time locus to a greater extent, as aforementioned delineation of the flowering time locus to be between 14.60 and 15.5 Mb based on the crossover site of IL013. Moreover, new recombinants with the crossover site between 14.5 and 15.5 Mb can be generated and employed to identify the lengths of introgressed chromosome segment to delineate the flowering time locus even greater using flowering time and genotypic data.

## Conclusion

Timing of flowering is very important for the breeders, agronomists, and ultimately for growers in deciding when to plant canola, as it helps minimize heat stress exposure during flowering time and effectively utilize soil moisture. In this study, a novel strategy combining both genetic and physical mapping methods was used to map a locus associated with flowering time in canola. This strategy, designated as “Marker-Assisted Lines Development with Series of Introgressed Chromosome Segments (MALDSICS)” is involved in three procedures: (1) developing introgression lines (ILs) carrying a series of introgressed chromosome A10 segments of Surpass 400 in various lengths using co-dominant polymorphic markers, (2) mapping flowering time locus between two adjacent markers by associating the phenotypic data with the introgressed chromosome segment, and (3) further delineating the map by identifying the crossover sites through genotyping. Using this strategy, we identified a 1 Mb region on chromosome A10 between 14.60 and 15.5 Mb of Surpass 400 that is linked to flowering time. In addition, we identified 5 genes within this region known to be involved in flowering time in Arabidopsis. These genes are likely candidates for the differences observed in flowering time of canola in this study. Although the co-dominant markers identified on chromosome A10 are very useful for marker assisted selection in breeding programs, they still need to be validated to other breeding populations or germplasm accessions of canola.

## Supplementary Information

Below is the link to the electronic supplementary material.

Supplemental Table S1. Genetic mapping using various molecular markers. (XLSX 111 KB)

Supplemental Table S2. Flowering time raw data. (XLSX 46 KB)

Supplemental Table S3. Statistical analysis. (XLSX 18 KB)

Supplemental Table S4. Genotyping of IL013. (XLSX 28 KB)

Supplemental Table S5. Genes located between 14.5-15.5 Mb chromosome region. (XLSX 44 KB)

## Data Availability

Data is contained within the article or supplementary material.
